# Anti-Diabetic Effect of Balanced Deep-Sea Water and Its Mode of Action in High-Fat Diet Induced Diabetic Mice

**DOI:** 10.3390/md11114193

**Published:** 2013-10-29

**Authors:** Byung Geun Ha, Eun Ji Shin, Jung-Eun Park, Yun Hee Shon

**Affiliations:** Bio-Medical Research Institute, Kyungpook National University Hospital, 50 Samduk 2ga Jung-gu, Daegu 700-721, Korea; E-Mails: hbg0281@hotmail.com (B.G.H.); shinej1204@naver.com (E.J.S.); eun5994@naver.com (J.-E.P.)

**Keywords:** deep-sea water, high-fat diet, diabetes, hyperglycemia, glucose tolerance

## Abstract

In this study, we investigated the effects of balanced deep-sea water (BDSW) on hyperglycemia and glucose intolerance in high-fat diet (HFD)-induced diabetic C57BL/6J mice. BDSW was prepared by mixing deep-sea water (DSW) mineral extracts and desalinated water to give a final hardness of 500–2000. Mice given an HFD with BDSW showed lowered fasting plasma glucose levels compared to HFD-fed mice. Oral and intraperitoneal glucose tolerance tests showed that BDSW improves impaired glucose tolerance in HFD-fed mice. Histopathological evaluation of the pancreas showed that BDSW recovers the size of the pancreatic islets of Langerhans, and increases the secretion of insulin and glucagon in HFD-fed mice. Quantitative reverse transcription polymerase chain reaction results revealed that the expression of hepatic genes involved in glucogenesis, glycogenolysis and glucose oxidation were suppressed, while those in glucose uptake, β-oxidation, and glucose oxidation in muscle were increased in mice fed HFD with BDSW. BDSW increased AMP-dependent kinase (AMPK) phosphorylation in 3T3-L1 pre- and mature adipocytes and improved impaired AMPK phosphorylation in the muscles and livers of HFD-induced diabetic mice. BDSW stimulated phosphoinositol-3-kinase and AMPK pathway-mediated glucose uptake in 3T3-L1 adipocytes. Taken together, these results suggest that BDSW has potential as an anti-diabetic agent, given its ability to suppress hyperglycemia and improve glucose intolerance by increasing glucose uptake.

## 1. Introduction

Type 2 diabetes is characterized by insulin resistance, hyperglycemia, and progressive beta-cell dysfunction, which leads to multiple diabetic complications like nephropathy, neuropathy, retinopathy, and ketoacidosis. Currently, the global prevalence of type 2 diabetes is rapidly increasing, resulting in significant financial and social burden worldwide. Numerous studies for the treatment of the type 2 diabetes have focused on maintaining normal levels of blood glucose by increasing glucose clearance in peripheral tissues, such as skeletal muscle and adipose tissue [1]. However, the complicated regulatory networks involved in the pathophysiology of diabetes have not been taken into account in previously identified drugs like the insulin-sensitizing biguanide (metformin) and thiazolidinedione (rosiglitazone and pioglitazone) drugs, which are targeted to a single molecule, such as adenosine monophosphate-dependent kinase (AMPK) [2] and peroxisome proliferator-activated receptor gamma (PPARγ) [3]. In addition, several clinical side effects including hypoglycemia, weight gain, edema, lactic acidosis, and gastrointestinal intolerance have been associated with the anti-diabetic properties of these drugs [4]. Therefore, developing novel safe drugs, with defined targets to treat the complex pathophysiology of diabetes, has become an urgent need. Recently, as an alternative strategy for developing effective and safe anti-diabetes drugs, many natural products, including crude extracts and compounds that have been isolated from natural resources, are being investigated to treat diabetes [5].

Deep-sea water (DSW), as a natural resource, is well-known, safe, stable, and in infinite supply compared to other natural products, and, as well, has high contents of unique minerals such as magnesium (Mg), calcium (Ca), potassium (K), zinc (Zn), and vanadium (V). Several studies of the physiological activity of DSW at a hardness of 1,200 ppm have suggested that DSW might be useful for the prevention of hyperlipidemia [6], hypertension [7,8], atopic eczema/dermatitis syndrome [9,10], and arteriosclerosis [11]. Moreover, Mg and Ca are well known for their anti-obesity and anti-diabetic effects [12,13,14]. Previously, *in vivo* [15] and *in vitro* [16] studies showed that DSW (hardness 1000 ppm) has anti-obesity and anti-diabetic properties, including inhibition of adipocyte differentiation and lipid accumulation, as well as anti-hyperglycemic activity. Drinking DSW (1% DSW (v/v)) can improve lipid metabolism and prevent atherosclerosis [17]. Total cholesterol levels were considerably decreased in the plasma of hypercholesterolemic subjects treated with DSW with high Mg concentrations (395 mg/L; hardness 1410 ppm) [18]. Therefore, it is worth studying the possibility that BDSW with high Mg and Ca content might be a new agent for treating or preventing metabolic disease. To date, effective DSW concentrations for treating or preventing metabolic disease and the detailed regulatory mechanisms by which DSW achieves its effects have not been well defined. Therefore, to investigate the effect of balanced deep-sea water (BDSW), of differing hardness, on obese type 2 diabetic mice, our study used BDSW ranging from relatively low (500 ppm) to high (2000 ppm) hardness.

In this study, we investigated the effect of BDSW on glucose uptake, and the mechanism of glucose uptake enhancement, using cultured 3T3-L1 adipocytes. To determine the effect of BDSW on the development of type 2 diabetes *in vivo*, we also examined fasting blood glucose levels and glucose tolerance in C57BL/6 mice that exhibited type 2 diabetes induced by high-fat diet feeding.

## 2. Results and Discussion

### 2.1. BDSW Suppresses Fasting Blood Glucose Levels and Improves Glucose Intolerance in High-Fat Diet (HFD)-Induced Diabetic Mice

**Figure 1:**
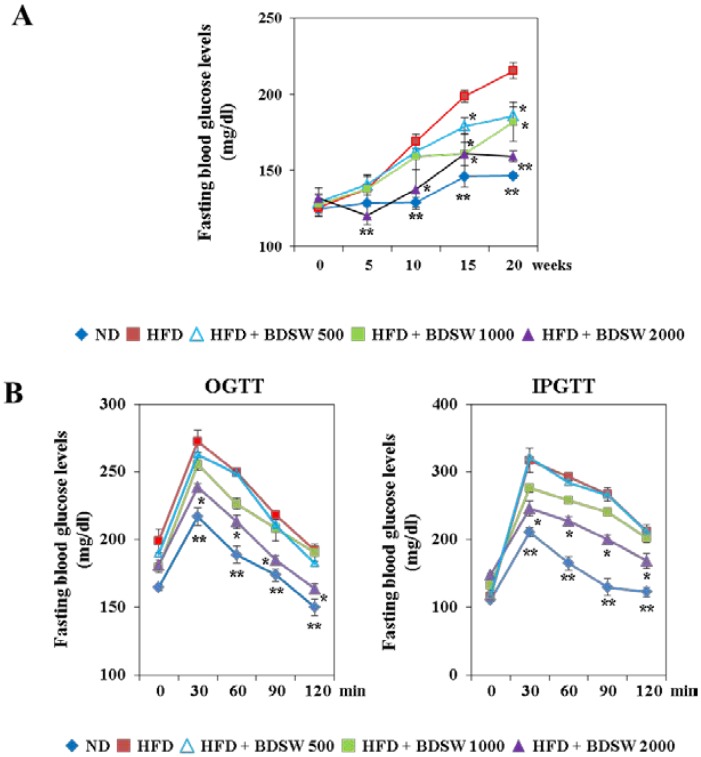
Effect of BDSW on fasting blood glucose levels (**A**) and glucose tolerance (**B**) in mice fed the ND, HFD, and HFD with BDSW for 20 weeks. Each value represents the mean ± SEM (*n* = 8 per group). * *P* < 0.05, ** *P* < 0.01: Significant difference *vs.* HFD-fed group. ND, normal diet; HFD, high-fat diet.

To determine the effect of BDSW on the development of diabetes, we investigated fasting blood glucose levels and glucose tolerance in HFD-induced diabetic mice. Chronic HFD-induced obesity is a well-established risk factor for the development of type 2 diabetes, and more than 80% of people with type 2 diabetes are overweight or obese [19,20]. Our findings also showed the characteristics of obese type 2 diabetes such as hyperglycemia, glucose intolerance, and increased weight gain in HFD-induced C57BL/6 mice. Prior to BDSW drinking, the zero-time fasting blood glucose levels did not differ between groups. During 20 weeks of BDSW drinking, BDSW attenuated the increase in the fasting blood glucose level in HFD-induced diabetic mice in a dose-dependent manner (Figure 1A). BDSW itself did not affect food intake. BDSW intake was similar between the HFD control group and the three BDSW groups. However, body weight gain was slightly decreased in the high hardness group (HFD + DSW 2000). This result seems to be caused by the decrease of intra-abdominal fat weight including mesenteric, epididymal, and perirenal depots (Table 1). Total triglycerides and cholesterol in liver and plasma were significantly higher in the HFD control group than in the ND group. Plasma and hepatic triglycerides and cholesterol decreased in a dose-dependent manner, but plasma insulin levels increased, in the BDSW groups (Table 1). To further evaluate the effect of BDSW on HFD-induced diabetic mice, we conducted the oral glucose tolerance test (OGTT) and intraperitoneal glucose tolerance test (IPGTT) at 19 weeks of BDSW drinking. The zero-time fasting blood glucose levels did not differ between groups. However, after glucose injection, BDSW improved the impaired glucose tolerance induced by HFD as measured by both glucose tolerance tests (Figure 1B).

**Table 1:** Effects of balanced deep-sea water (BDSW) on body weight, food intake, serum/liver triglycerides and total cholesterol levels, and serum insulin levels in HFD-induced diabetic mice. Body weight, food intake, tap water and BDSW intake were measured once every three days for 20 weeks. The levels of triglycerides and total cholesterol in serum and liver, and insulin in serum were measured at 20 weeks of BDSW drinking. Each value represents the mean ± SEM (*n* = 8 per group). * *P* < 0.05, ** *P* < 0.01: Significant difference *vs.* HFD group. ND, normal diet; HFD, high-fat diet.

Measurement	ND	HFD	HFD + BDSW 500	HFD + BDSW 1000	HFD + BDSW 2000
Initial body weight (g)	18.9 ± 0.4 **	19.2 ± 0.3	18.7 ± 0.3	18.9 ± 0.1	19.0 ± 0.1
Final body weight (g)	25.3 ± 0.1 **	35.9 ± 0.6	33.7 ± 0.3	34.1 ± 0.4	33.2 ± 0.1 *
Weight gain (g/20 weeks/mouse)	6.3 ± 1.0 **	16.74 ± 1.7	15.1 ± 2.5	15.2 ± 1.6	14.1 ± 1.4 *
Intra-abdominal fat weight (g/100 g body weight)	1.8 ± 0.1 **	8.2 ± 0.2	8.1 ± 0.3	8.02 ± 0.1	7.6 ± 0.1 *
Food intake (g/day/mouse)	2.5 ± 0.1 **	2.2 ± 0.1	2.3 ± 0.12	2.3 ± 0.1	2.2 ± 0.1
Serum triglycerides	45.6 ± 1.7 **	81.2 ± 6.4	65.9 ± 9.1 *	61.5 ± 8.5 *	49.6 ± 7.5 **
Total cholesterol (mg/dL)	78.9 ± 3.8 **	142.3 ± 4.3	131.52 ± 2.7 *	133.8 ± 2.7 *	126.7 ± 3.6 *
Liver triglycerides	136.57 ± 1.6 **	246.5 ± 19.9	221.8 ± 23.6	195.8 ± 6.5 *	187.5 ± 7.6 *
Total cholesterol (mg/g liver)	99.8 ± 3.6 **	147.7 ± 5.1	149.2 ± 5.4	132.7 ± 7.1 *	129.6 ± 5.1 *
Tap water & BDSW intake (mL/day/mouse)	4.8 ± 0.1 **	3.9 ± 0.1	4.1 ± 0.1	4.2 ± 0.1	4.3 ± 0.1
Serum insulin level (ng/mL)	4.2 ± 0.8	3.6 ± 0.1	3.7 ± 0.3	5.4 ± 0.8 *	6.3 ± 0.8 *

### 2.2. Effect of BDSW on the Levels of Adiponectin, Leptin, and Insulin in the Plasma and Pancreas Functions of HFD-Induced Diabetic Mice

**Figure 2:**
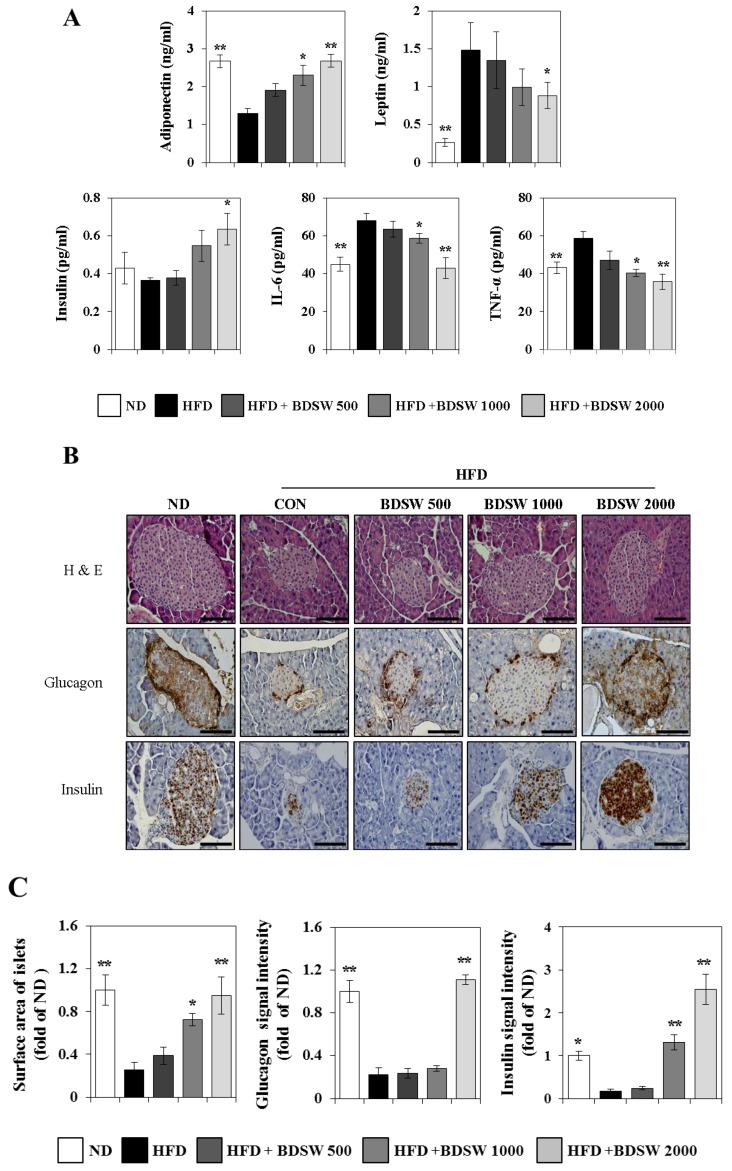
Effects of BDSW on adipokines and cytokines levels, in plasma (**A**) and morphological changes in pancreas (**B**) of mice fed the ND, HFD, and HFD with BDSW for 20 weeks. (**C**) Surface area of the islets of Langerhans and intensities of staining signals for insulin and glucagon. Representative hematoxylin & eosin, glucagon, and insulin staining of the islets of Langerhans are shown at 400× magnification. Scale bar, 50 μM. Each value represents the mean ± SEM (*n* = 8 per group). * *P* < 0.05, ** *P* < 0.01: Significant difference *vs.* HFD-fed group. ND, normal diet; HFD, high-fat diet.

To investigate the regulatory mechanism of hyperglycemia and insulin resistance in greater detail, we investigated the levels of the major insulin-sensitizing adipokines, adiponectin and leptin, and that of insulin, in the plasma of HFD-induced diabetic mice. Adipokines are polypeptides that are secreted from adipocytes and function as classic circulating hormones to communicate with other organs including brain, liver, muscle, the immune system, and adipose tissue itself. The dysregulation of adipokines has been implicated in obesity, type 2 diabetes, and cardiovascular disease [21]. Adiponectin and leptin are well known as anti-obesity and anti-diabetic adipokines. Adiponectin is an insulin-sensitizing hormone that exhibits direct anti-diabetic, anti-atherogenic, and anti-inflammatory potential [22] in addition to modulating a number of metabolic processes, including glucose and fatty acid metabolism [23]. Leptin represses food intake and promotes energy expenditure. Predictably, animals and humans that have mutations in either leptin or leptin receptors are obese [24]. BDSW increased adiponectin and insulin levels in a dose-dependent manner, but decreased leptin levels (Figure 2A). These results seem to be caused by hyperleptinemia in diet-induced obesity and the failure of pancreatic function, including insulin production, in beta cells. Moreover, BDSW dose-dependently decreased interleukin-6 (IL-6) and tumor necrosis factor-α (TNF-α) levels, which are known as proinflammatory cytokines. Particularly noteworthy is the high leptin level in HFD-fed mice. Chronic HFD-induced high leptin levels have been observed to be accompanied by a reduction of central leptin sensitivity [25]. Recently, inflammatory responses in adipose tissue have been shown as a major mechanism to induce peripheral tissue insulin resistance [26]. Although adiponectin and leptin regulate feeding behavior and energy expenditure, these adipokines are also involved in the regulation of inflammatory responses. Adipose tissue secretes various pro- and anti-inflammatory adipokines such as TNF-α and IL-6 to modulate inflammation and insulin resistance. In obese humans and rodent models [27], the expression of pro-inflammatory adipokines is enhanced to induce insulin resistance. These pro-inflammatory adipokine levels also were decreased by BDSW. Collectively, these findings suggest that BDSW regulates the physiological and molecular functions of adipokines in HFD-fed mice. On the other hand, histological assay of the pancreas revealed that BDSW improves the architecture of the pancreatic islets of Langerhans and the levels of glucagon from alpha cells and insulin from beta cells in the HFD-fed group. Glucagon levels in mice fed HFD with BDSW (hardness 2000 ppm) were similar to that of the ND-fed group. On the other hand, insulin levels were remarkably increased in mice fed HFD with BDSW (hardness 2000 ppm) (Figure 2B). The increase in insulin levels in the BDSW group was associated with improved pancreatic function. However, more detailed are needed to elucidate the regulatory mechanism in BDSW-induced insulin production. Omar *et al.* [28] showed that HFD (60% fat) and HFD (58% fat)-high sucrose diet (HSD) result in different models with respect to the degree of insulin resistance and beta cell dysfunction induced in a relatively short period of time. HFD for eight weeks elevated the basal insulin level, but the acute insulin response and insulin secretion were not changed. However, our results showed that HFD for 20 weeks decreased insulin levels owing to pancreatic dysfunction, including reduced alpha and beta cell volume and function. BDSW at a hardness of 2000 ppm remarkably increased insulin level in HFD-fed mice and recovered glucagon levels, similar to ND-fed mice (Figure 2C). These results appear to be caused by different HFD levels and feeding periods.

### 2.3. Effect of BDSW on the Expressions of Genes Related to Glucose Homeostasis in Livers and Muscles of HFD-Induced Diabetic Mice

**Figure 3:**
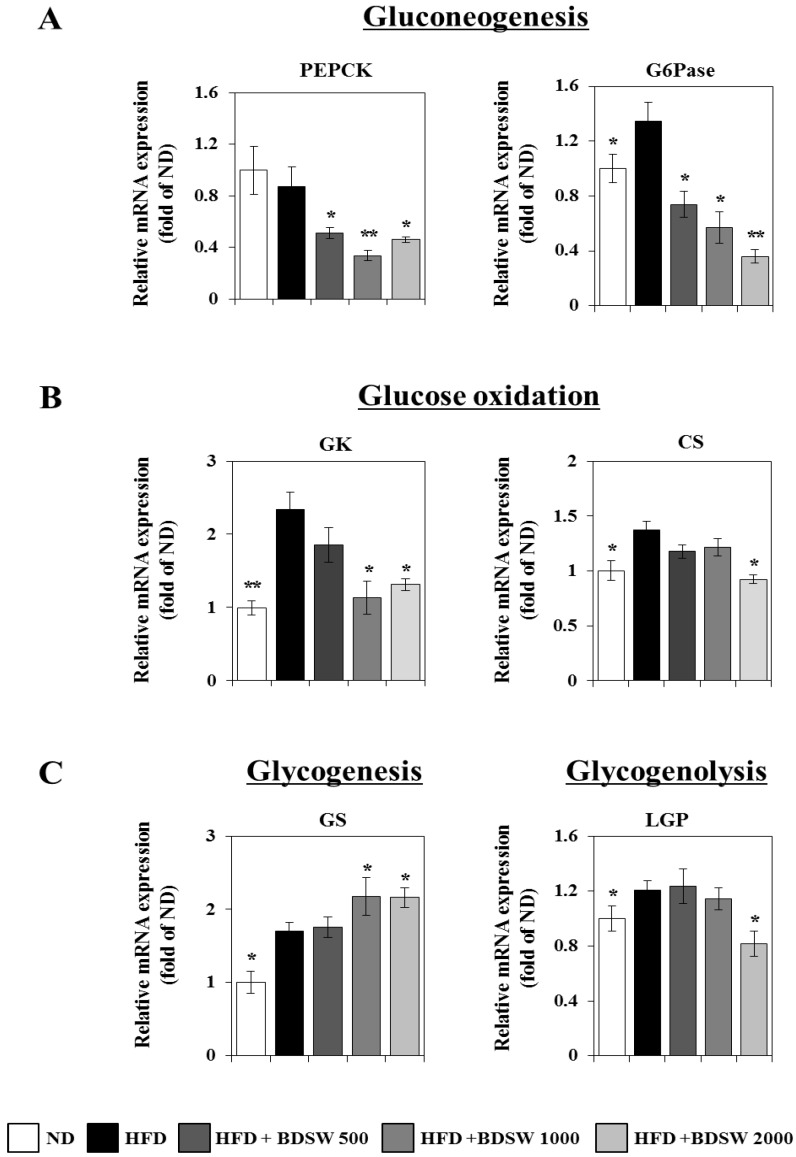
Effects of BDSW on the expression of genes involved in gluconeogenesis (**A**), glycogen metabolism (**B**), and glucose oxidation (**C**) in the livers of mice fed the ND, HFD, and HFD with BDSW for 20 weeks. Each value represents the mean ± SEM (*n* = 8 per group) in three independent experiments. * *P* < 0.05, ** *P* < 0.01: Significant difference *vs.* HFD-fed group. ND, normal diet; HFD, high-fat diet.

**Figure 4:**
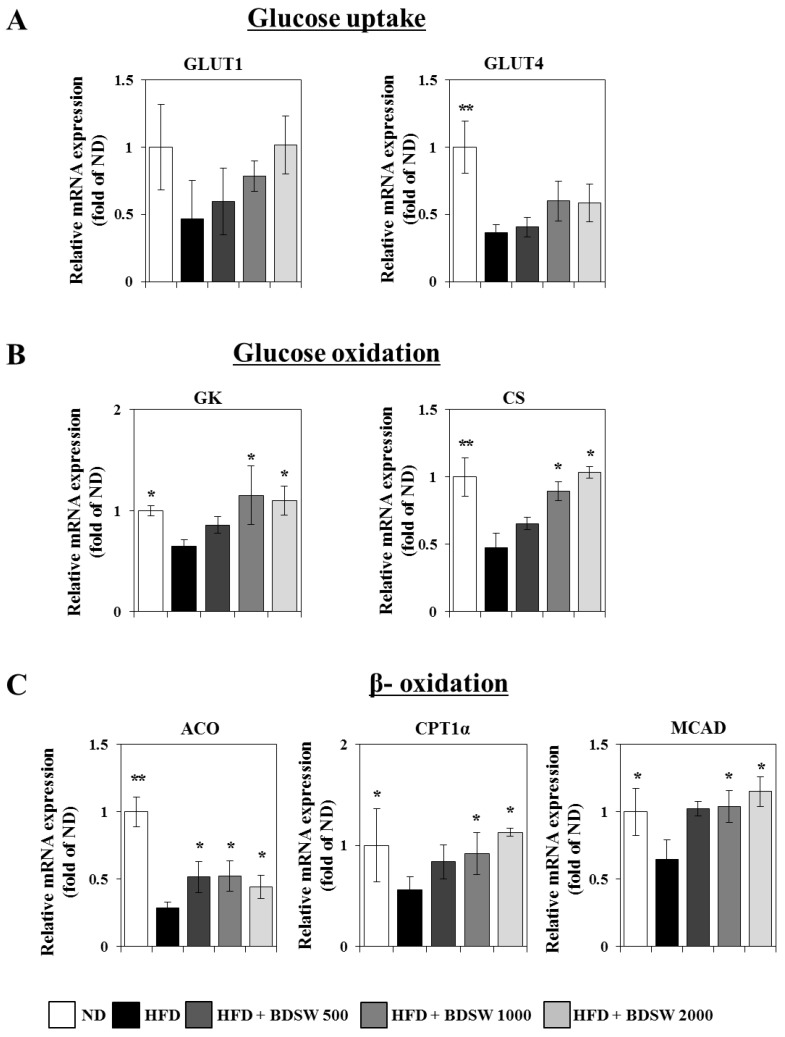
Effects of BDSW on the expression of genes involved in glucose uptake (**A**), glucose oxidation (**B**), and β-oxidation (**C**) in the muscles of mice fed the ND, HFD, and HFD with BDSW for 20 weeks. Each value represents the mean ± SEM (*n* = 8 per group) in three independent experiments. * *P* < 0.05, ** *P* < 0.01: significant difference *vs.* HFD-fed group. ND, normal diet; HFD, high-fat diet.

Blood glucose levels are regulated by the coordinated actions of various organs such as liver, muscle, pancreas, fat, intestine, and brain [29]. Inputs to blood glucose levels include absorption from the intestine and release from the liver. The latter occurs by breakdown of preformed glycogen (glycogenolysis), as well as the generation of glucose from non-carbohydrate carbon substrates (gluconeogenesis), and both processes are inhibited by insulin. Glucose is removed from blood by uptake into virtually all cell types, but most importantly into muscle and adipose tissue, which requires insulin. We next investigated whether BDSW influences the glucose homeostasis metabolism including gluconeogenesis, glycogenesis, glycogenolysis, and glucose oxidation in livers, as well as glucose uptake, glucose oxidation, and β-oxidation in skeletal muscle. Quantitative real-time PCR analysis showed that BDSW remarkably down-regulated the expression of phosphoenolpyruvate carboxykinase (PEPCK) and glucose 6-phosphatase (G6Pase) for gluconeogenesis (Figure 3A), glucokinase (GK) and citrate synthase (CS) for glucose oxidation (Figure 3B), and liver glycogen phosphorylase (LGP) for glycogenolysis (Figure 3C). However, the expression of glycogen synthase (GS) for glycogenesis was up-regulated (Figure 3C). On the other hand, in skeletal muscle, BDSW up-regulated the expression of glucose transporter 1 (GLUT1) and glucose transporter 4 (GLUT4) for glucose transport (Figure 4A), GK and CS for glucose oxidation (Figure 4B), and acyl-CoA oxidase (ACO), carnitine palmitoyl transferase 1α (CPT1α), and mitochondrial medium-chain acyl-CoA dehydrogenase (MCAD) for β-oxidation (Figure 4C). In addition, BDSW increased the expression of sirtuin family proteins such as SIRT1, SIRT4, and SIRT6 (Supplemental Figure S1). Canto *et al.* [30] showed that AMPK enhances SIRT1 activity by increasing cellular NAD(+) levels, resulting in the deacetylation and modulation of the activity of downstream SIRT1 targets including the PPARγ coactivator 1α (PGC-1α) and the forkhead box O1 (FOXO1) and FOXO3a transcription factors. The AMPK-induced SIRT1-mediated deacetylation of these targets explains many of the convergent biological effects of AMPK and SIRT1 on energy metabolism [31]. Some reports regarding the effect of natural products on the glucose uptake mechanism [32,33] support our observation that stimulation of glucose uptake by BDSW occurs through AMPK. Therefore, BDSW seems to regulate several genes related to energy metabolism including glucose, lipid, and fatty acid metabolism through the AMPK/Sirtuin1 pathways, although we did not confirm the activity of SIRT1, SIRT4, SIRT6 by BDSW treatment. These findings suggest that BDSW regulates the blood glucose level through inhibition of glucose production and enhancement of glucose uptake mediated by gene regulation by the AMPK/Sirtuin pathway.

### 2.4. BDSW Stimulates Glucose Uptake by 3T3-L1 Adipocytes in a Dose-Dependent Manner

**Figure 5:**
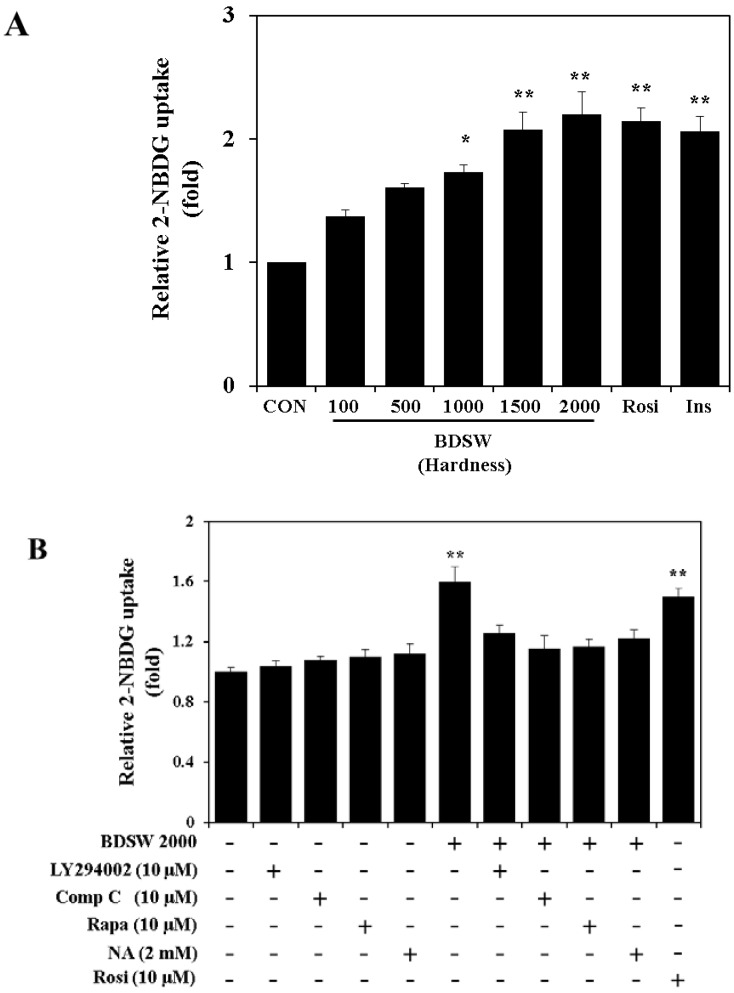
Effects of BDSW on 2-NBDG uptake in mature 3T3-L1 adipocytes. For the glucose uptake assay at different levels of BDSW hardness (**A**), 3T3-L1 adipocytes were preincubated in DMEM without glucose for 1 h. They were then incubated in DMEM containing BDSW at different levels of hardness, or 10 μM rosiglitazone (Rosi), and 100 nM insulin (Ins) with 20 μM 2-NBDG for 1 h. For the glucose uptake assay using several inhibitors (**B**), after preincubation in DMEM without glucose in the presence or absence of 10 μM LY294002, 10 μM compound C (Comp C), 10 μM rapamycin (Rapa), 2 mM nicotinamide (NA), and 10 μM rosiglitazone (Rosi) for 1 h, the cells were incubated in DMEM containing BDSW of hardness 2000 ppm only, with or without inhibitors with 20 μM 2-NBDG for 1 h. After incubation was completed, cells were lysed, and then the glucose uptake was measured using a fluorometer. Each value represents the mean ± SEM for six wells in three independent experiments. * *P* < 0.05, ** *P* < 0.01: Significant difference *vs.* HFD-fed group. ND, normal diet; HFD, high-fat diet; Comp C, compound C; Rapa, rapamycin; NA, nicotinamide; Rosi, rosiglitazone.

Based on our *in vivo* findings, to determine whether BDSW directly stimulates glucose uptake, we performed a glucose uptake assay to measure 2-NBDG uptake in mature 3T3-L1 adipocytes. Insulin-stimulated glucose uptake by adipocytes and skeletal muscle play an important role in the maintenance of whole-body glucose homeostasis. GLUT4 is the main glucose transporter isoform expressed in adipocytes and skeletal muscle that mediates glucose uptake in response to hormones such as insulin. Insulin acutely stimulates glucose uptake by GLUT4 translocation to the cell surface of adipocytes and skeletal muscle [34]. BDSW significantly increased glucose uptake in a dose-dependent manner (Figure 5A). The increased level of glucose uptake induced by BDSW at a hardness of 2000 ppm was similar to the levels induced by rosiglitazone and insulin, which are well known as agonists of glucose uptake in mature adipocytes. To explore the regulatory mechanisms of BDSW-promoted glucose uptake, we next examined the effects of several inhibitors on BDSW-promoted glucose uptake, namely, LY294002 (a potent inhibitor of phosphoinositide 3-kinase; PI3-K), compound C (an ATP-competitive inhibitor of AMPK), rapamycin (an inhibitor of mammalian target of rapamycin; mTOR), and nicotinamide (a Sirt1 inhibitor). The promotion of glucose uptake by BDSW was completely inhibited by treatment with LY294002, compound C, rapamycin, and nicotinamide (Figure 5B). These results suggest that the stimulatory effect of BDSW on glucose uptake is dependent on the PI3-K, AMPK, mTOR, and Sirt1 pathways.

### 2.5. BDSW Activates the PI-3K and AMPK Signaling Pathways

**Figure 6:**
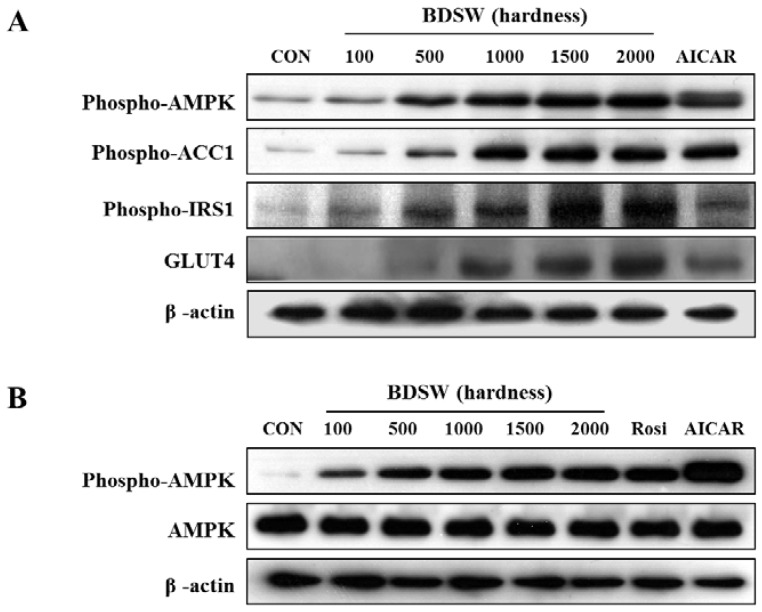
Effects of BDSW on the phosphorylation of AMPK for 3T3-L1 adipocyte differentiation (**A**), or in mature adipocytes (**B**). 3T3-L1 pre- and mature adipocytes were treated in media containing BDSW at different levels of hardness for eight days or 1 h, respectively, lysates (20 μg) were subjected to SDS-PAGE and western blotting analyses using anti-phospho-AMPK, anti-phospho-ACC1, and anti-phospho-IRS-1, anti-GLUT4 antibodies. β-actin was used as a loading control.

To determine more precisely the regulatory mechanism by which BDSW stimulates glucose uptake, we investigated signal molecules related to glucose uptake in 3T3-L1 preadipocytes and adipocytes. BDSW specifically stimulated in a dose-dependent manner the phosphorylation of AMPK and ACC1, a substrate of AMPK, for adipocyte differentiation (Figure 6A). AMPK is a major cellular energy sensor and a master regulator of metabolic homeostasis like glucose, carbohydrate, lipid, and protein metabolism, and is activated by decreases in the cellular energy state as reflected by an increased AMP/ATP ratio [35]. AMPK is also known as an attractive therapeutic target for metabolic diseases, including diabetes [36]. Moreover, BDSW increased the phosphorylation of IRS-1 and the expression of GLUT4 related to glucose uptake through the insulin-signaling pathway (Figure 6A). Moreover, AMPK phosphorylation was increased in mature adipocytes (Figure 6B). These results suggest that BDSW increases not only the activity of signal molecules related to the insulin signaling pathway such as IRS-1 and AMPK, but also the expression of GLUT4, which is involved in adipocyte function for glucose uptake.

### 2.6. BDSW Specifically Stimulates AMPK Phosphorylation and Improves Impaired AMPK Phosphorylation in the Muscles and Livers of HFD-Induced Diabetic Mice

**Figure 7:**
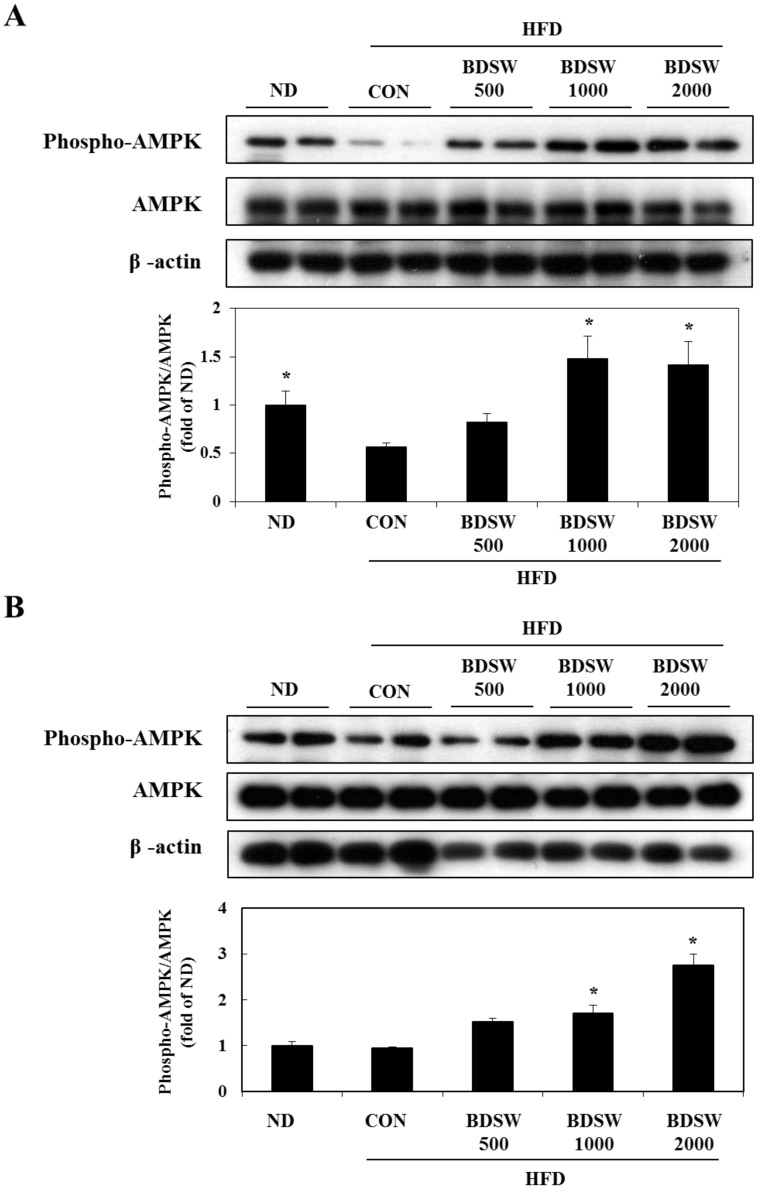
Effects of BDSW on the phosphorylation of AMPK in muscles (**A**) and livers (**B**) of mice fed the ND, HFD, and HFD with BDSW for 20 weeks. Each value represents the mean ± SEM (*n* = 8 per group). * *P* < 0.05: Significant difference *vs.* CON of HFD fed group. ND, normal diet; HFD, high fat diet.

To determine whether BDSW stimulates AMPK phosphorylation *in vivo*, we investigated the phosphorylation of AMPK in the muscle and livers of mice fed ND, HFD, and HFD with BDSW. The phosphorylation of AMPK in muscle of HFD-fed mice was decreased considerably. However, mice fed HFD with BDSW for 20 weeks showed about three-fold increased phosphorylation of AMPK in comparison with HFD-fed mice (Figure 7A). On the other hand, in liver, the phosphorylation of AMPK in HFD-fed mice was similar to that of ND fed mice. Mice fed HFD with BDSW showed notably increased phosphorylation of AMPK in a dose-dependent manner. Therefore, these results suggested that BDSW may regulate glucose and lipid metabolism by AMPK pathway in muscles and livers. To date, two AMPK kinases have been characterized: the tumor suppressor LKB1 [37] and Ca^2+^/Calmodulin-dependent protein kinase kinase β (CaMKKβ) [38]. LKB1 is ubiquitously expressed and appears to be the kinase that is most important for the activation of AMPK in response to cellular stress in liver and skeletal muscle. In contrast to LKB1, the activation of AMPK by CaMKKβ does not require alteration of the ATP:AMP ratio, but rather occurs in response to an increase in intracellular Ca^2+^. The expression pattern of CaMKKβ in cells and tissues is limited compared to that of LKB1 and is the highest in multiple regions of the brain [39]. A previous study [40] has demonstrated that a CaMKKβ-AMPK signaling pathway in the hypothalamus is important in the regulation of appetite. This led us to propose that activation of AMPK by LKB1 may be most critical at the cellular level, whereas regulation of AMPK by CaMKKβ may be more important at the whole body level [41].

## 3. Experimental Section

### 3.1. Materials

3T3-L1 cells were purchased from the Korean Cell Line Bank (Seoul, Korea; KCLB No. 10092.1). Male C57BL/6J mice (5 weeks of age) were obtained from Charles River Japan (Charles River Japan, Kanagawa, Japan). The following items were purchased from the cited commercial sources: 5-aminoimidazole-4-carboxamide 1-β-d-ribofuranoside, acadesine, *N*1-(β-d-ribofuranosyl)-5-aminoimidazole-4-carboxamide (AICAR) and 5-[[4-[2-(methyl-2-pyridinylamino)ethoxy]phenyl]methyl]-2,4-thiazolidinedione (rosiglitazone), 3,3-diaminobenzidine tetrahydrochloride (DAB) from Sigma-Aldrich Co. LLC. (St. Louis, MO, USA); Glucose, Cleantech TG-S, and ASAN SET Total-Cholesterol assay kit from Asan Pharmaceutical Co. Ltd. (Seoul, Korea); Ultra Sensitive Mouse Insulin ELISA kit from Crystal Chem Inc. (Downers Grove, IL, USA); Mouse Adiponectin/Acrp30 and Leptin ELISA kit from R&D Systems, Inc. (Minneapolis, MN, USA); Mouse IL-6 and TNF-ELISA kit from Biolegend, Inc. (San Diego, CA, USA); anti-phospho AMPK, anti-AMPK, anti-phospho acetyl co-A carboxylase 1 (ACC1), anti-phospho insulin receptor substrate 1 (IRS1), and anti-glucose transporter 4 (GLUT4) antibodies from Cell Signaling Technology (Danvers, MA, USA); guinea pig anti-human insulin, and rabbit anti-glucagon from Merk Millipore Co. (Billerica, MA, USA); peroxidase-conjugated AffiniPure goat anti-rabbit Ig (H + L) from proteintech group Inc. (Chicago, IL, USA); anti-β-actin, horseradish peroxidase (HRP)-conjugated anti-mouse IgG, and anti-rabbit IgG-HRP antibodies from Santa Cruz Biotechnology Inc. (Santa Cruz, CA, USA); ECL Plus Western Blotting Substrate from Pierce Biotechnology (Rockford, IL, USA); Trizol and 2-(*N*-(7-nitrobenz-2-oxa-1,3-diazol-4-yl)amino)-2-deoxyglucose (2-NBDG) from Invitrogen Life Technologies (Carlsbad, CA, USA); PrimeScript™ 1st strand cDNA Synthesis Kit from Takara Bio Inc. (Shiga, Japan); FastStart Universal SYBR Green Master from Roche Applied Science (Basel, Switzerland); Phosphatase Inhibitor Cocktail and Protease Inhibitor Cocktail solutions from GenDEPOT (Barker, TX, USA).

### 3.2. The Preparatioin of BDSW

Original DSW that had been pumped up from a depth of 0.5 km and a distance of 6.7 km off Oho-Ri, Goseong (Gangwon-Do, Korea) was filtered using a microfilter system (Synopex INC, Pohang, Korea) to remove phytoplankton and marine microorganisms. The filtered DSW was passed through a reverse osmosis membrane (Vontron Technology Co., Ltd., Beijing, China), and DSW mineral extracts and desalinated water were obtained. Next, the DSW mineral extracts and the desalinated water were mixed to prepare BDSW having an Mg:Ca ratio of 3:1. The BDSW was serially diluted with regard to hardness. BDSW used in this study did not include any peptides or organic molecules. In this study, we defined the hardness of BDSW by focusing on the concentrations of Mg and Ca. The hardness values were calculated according to the following equation: Hardness = Mg (mg/L) × 4.1 + Ca (mg/L) × 2.5. Supplemental Table S1 shows the mineral content of the original and BDSW samples with a hardness of 4531 ppm. All of the BDSW used in this study was sterilized by passing it through a 0.2-μM bottle-top filter (Fisher Scientific Inc., IL, USA).

### 3.3. Animals and Diets

All animal experiments were conducted in accordance with the guidelines established by the Animal Ethics Committee of Kyungpook National University, and the protocols were approved by this committee (Approval No. KNU 2012-88). To determine the effect of BDSW on fasting blood glucose levels, C57BL/6J mice were used as the animal model. All of the experiments in this study were conducted on 6–26-week-old male littermates. The mice were housed in an air-conditioned room with a temperature of 22 ± 2 °C, a relative humidity of 40% ± 5% and an 8:00–20:00 light cycle. All the mice were maintained on a stock CRF-1 pellet diet (Oriental Yeast Co., Tokyo, Japan). After preliminary feeding for 1 week, the C57BL/6J mice (6 weeks of age) were divided into 5 groups with similar fasting blood glucose levels and body weights. The C57BL/6J mice in each of the 5 groups (eight mice per group) were given either the normal diet (ND) (D12450B, 10% kcal% fat; Research diet, New Brunswick, NJ, USA), HFD (D12451, 45% kcal% fat; Research diet, New Brunswick, NJ, USA), or HFD with BDSW of differing hardness (500, 1000, and 2000 ppm) for 20 weeks. The weight and intake of water and food were measured every 3 days. Water and food were always available, and after the mice were deprived of their diet for 4 h, blood was collected every week to determine fasting blood glucose levels as described below. At the end of the feeding period, blood was collected from the tail vein, followed by exsanguination under anesthesia with zoletil (Virbac S.A., Carros, France). To determine the contribution of abdominal fat to total body fat, three intra-abdominal fat pads (epididymal, perirenal, and mesenteric) were excised and weighed upon sacrifice.

### 3.4. Determination of Blood Glucose Level

To determine fasting blood glucose levels, mice were deprived of their diet until blood collection from the tail vein 4 h later but allowed free access to water. Blood (10 μL) was added to water (30 μL), 20% (w/v) trichloroacetic acid aqueous solution (40 μL) was added, and test tubes containing the mixture were kept in ice-cold water. The mixture was then centrifuged at 12,000 × *g* and 4 °C for 10 min. The resultant supernatant (10 µL) was subjected to glucose determination using the Glucose Test Kit (Asan Pharmaceutical Co. Ltd., Seoul, Korea) and the absorbance at 505 nm was measured using a spectrophotometer (VersaMax microplate reader; Sunnyvale, CA, USA).

### 3.5. Cell Culture and Adipocyte Differentiation

3T3-L1 cells were maintained in DMEM containing 10% calf serum and antibiotics (penicillin-streptomycin). To induce differentiation, 2 days post-confluent 3T3-L1 cells were incubated in differentiation induction medium containing DMEM with 10% FBS, 0.5 mM IBMX, 1 mM dexamethasone, and 10 μg/mL insulin for 2 days. Two days after MDI induction, the medium was changed to medium containing 10 μg/mL insulin. The cells were then maintained in DMEM with 10% FBS for an additional 2 days until fully differentiated.

### 3.6. Triglyceride and Cholesterol Assays of the Liver and Serum

From each mouse, 0.2 g of liver tissue was homogenized in a Polytron homogenizer with 3 mL chloroform/methanol (2:1, v/v). Following, fresh chloroform/methanol (3 mL; 2:1, v/v) was added, homogenates were vortexed for 10 min and filtered with Advantec filter paper #131(Toyo Roshi Kaisha, Ltd., Tokyo, Japan). A sample (200 µL) of the organic phase was mixed with Triton X-100 (final concentration, 0.1%). After evaporation of the organic solvents, the lipid in the detergent phase was used to measure triglycerides and total cholesterol content. Using the Cleantech TG-S, and ASAN SET Total-Cholesterol assay kit as described by the manufacturer’s instructions (Asan Pharmaceutical Co. Ltd., Seoul, Korea), the amount of triglyceride and total cholesterol in liver (5 µL) and serum (2 µL) samples were determined by the absorbances at 600 nm using a microplate reader (Model AD200; Beckman Coulter, Brea, CA, USA).

### 3.7. Oral Glucose Tolerance Test (OGTT) and Intraperitoneal Glucose Tolerance Test (IPGTT)

The OGTT and IPGTT were performed at 19 weeks after BDSW drinking. Briefly, a ND-fed group, a HFD-fed group, and 3 groups that received HFD with BDSW (hardness 500, 1000, and 2000 ppm) were deprived of their diet but allowed free access to water. After fasting for 6 h or 15 h, blood was collected from the tail veins of all mice (0 min). Immediately after blood collection, all mice received oral or intraperitoneal injection of glucose (0.2 g/100 g body weight). Blood samples were successively collected at the indicated time intervals (0, 30, 60, 90, and 120 min), and blood glucose levels were determined as mentioned above.

### 3.8. Histopathological Analysis of Pancreas

Pancreas tissues were fixed in 4% formalin over 24 h. Fixed tissues were processed routinely for paraffin embedding. Insulin and glucagon were detected in the 5-μM pancreatic sections by guinea pig anti-human insulin diluted 1/100 (Millipore) and rabbit anti-glucagon diluted 1/3000 (Millipore Corp., Billerica, MA, USA), respectively, followed by incubation with peroxidase-conjugated AffiniPure goat anti-rabbit Ig (H + L) diluted 1/100 (Proteintech Group Inc., Chicago, USA). 3,3-diaminobenzidine tetrahydrochloride (DAB: Sigma-Aldrich, St. Louis, MO, USA) was applied to the sections as the substrate for peroxidase. The sections were counterstained with hematoxylin. Image acquisition was performed using an optical microscope (Nikon Eclipse 80i; Tokyo, Japan) with a magnifying power of 400. For size determination of islets of Langerhans, the surface area of islets of Langerhans was traced manually and analyzed using Image J software (*n* = 8 per group). The intensities of immunostained signals for insulin and glucagon proteins were measured as the optical mean density (*n* = 8 per group). All quantitative measurements were performed using Image J software.

### 3.9. Determination of Glucose Uptake by 3T3-L1 Adipocytes

Glucose uptake by mature 3T3-L1 adipocytes was measured using 2-NDBG as previously described [42]. Mature 3T3-L1 adipocytes differentiated on 96-well black culture plates (Greiner Bio-One, CELL STAR 96W Plate, NC, USA) were washed with phosphate-buffered saline (PBS). The cells were starved with serum-free low-glucose DMEM for 1 h before incubating with DMEM containing different levels of BDSW hardness and 20 μΜ 2-NBDG in the presence or absence of 10 μΜ rosiglitazone and 100 nM insulin. After incubation for 1 h, cells were washed with PBS and incubated with lysis buffer (0.1 M potassium phosphate, 1% Triton X-100, pH 10) for 10 min with shaking. Subsequently, dimethyl sulfoxide (DMSO) was added with shaking for 10 min. The fluorescence signal was measured with a microplate reader (FLUOstar OPTIMA, BMG LABTECH, Germany) at excitation and emission wavelengths of 466 nm and 540 nm, respectively.

### 3.10. Western Blot Analysis

Cells and tissues were washed with ice-cold PBS, and lysed in RIPA buffer (50 mM NaCl, 10 mM Tris, 0.1% sodium dodecyl sulfate (SDS), 1% Triton X-100, 0.1% sodium deoxycholate, 5 mM EDTA, 1 mM Na3VO4, pH 7.4). Total protein (40 μg) was separated by SDS-polyacrylamide gel electrophoresis and transferred to a nitrocellulose membrane (Whatman, Dassel, Germany). The membrane was blocked with 5% skim milk for 1 h and incubated with primary antibodies (diluted 1:1000) overnight at 4 °C. After washing with Tris-buffered saline containing 0.1% Tween-20, the membrane was incubated with HRP-conjugated secondary antibodies (diluted 1:3000) for 1 h at room temperature. Antibody binding on the nitrocellulose membrane was detected with an enhanced chemiluminescence solution (Amersham Bioscience, Buckinghamshire, UK) and radiography. The intensity of each band was analyzed with a Lumino image analyzer (Model LAS-4000 Mini; Fujifilm, Tokyo, Japan) coupled with image analysis software (Multi Gauge Ver. 3.0; Fujifilm).

### 3.11. Quantitative Reverse Transcription Polymerase Chain Reaction (RT-PCR) Analysis

Total RNA was isolated from 3T3-L1 cells, muscles, and livers in mice using Trizol (Invitrogen, Carlsbad, CA, USA), and complementary deoxyribonucleic acid (cDNA) was synthesized using a PrimeScript™ 1st strand cDNA synthesis kit (Takara Bio Inc., Shiga, Japan) according to the manufacturer’s instructions. Real-time PCR was performed in triplicate using a FastStart SYBR Green Master (Roche Diagnostics, Mannheim, Germany) in an ABI Prism 7300 Sequence Detection System (Applied Biosystems, Foster City, CA, USA). The expression levels of the target genes relative to that of the endogenous reference gene actin were calculated using the delta cycle threshold method. Data were the mean ± SEM calculated from three independent experiments in triplicate. The results were expressed as fold increase relative to the ND group (=1.0) after normalization using the expression level of the actin gene. The primer sequences are listed in Supplemental Table S2.

### 3.12. Statistical Analysis

All experimental results were compared by one-way analysis of variance (ANOVA) using the Statistical Package for the Social Sciences (SPSS, ver. 11.0, LEAD Technologies, Inc., Charlotte, NC, USA) program, and the data were expressed as means ± SEM. Group means were considered to be significantly different at *p* < 0.05 as determined by the technique of protective least significant difference when ANOVA indicated an overall significant treatment effect of *p* < 0.05.

## 4. Conclusions

In conclusion, we investigated the anti-diabetic potential of BDSW in HFD-induced diabetic mice. Our findings indicate that the inhibition of hyperglycemia and improvement of glucose intolerance by BDSW may be mediated, at least in part, by the downregulation of genes related to gluconeogenic, glycogenolytic, and glucose oxidation processes in the liver, as well as the upregulation of genes related to glucose uptake in skeletal muscle. Additionally, BDSW restored AMPK activation. Therefore, we suggest that BDSW, a novel activator for glucose uptake, is a valuable agent for treating or preventing diabetes.

## Supplementary Files

Supplementary File 1 Supplementary Information (PDF, 45 KB)
