# Supplementary material for: Anti-Diabetic Effect of Balanced Deep-Sea Water and Its Mode of Action in High-Fat Diet Induced Diabetic Mice
Source: Mar Drugs. 2013 Oct 29;11(11):4193–212. doi: 10.3390/md11114193 (PMC3853723; doi:10.3390/md11114193)

Supplementary Information

Table S1. Mineral content of original DSW and balanced DSW used in this study (Ex. Hardness 4531).

Major Elements	Balanced DSW (mg/kg)	Original DSW (mg/kg)
Ca	294	417
Mg	922	1299
Na	51	10,794
Cl	596	18,607
SO_4	59	2624

Table S2. Primers for quantitative real-time polymerase chain reaction analysis.

	Forward	Reverse
**PEPCK**	CTGGCACCTCAGTGAAGACA	TCGATGCCTTCCCAGTAAAC
**G6Pase**	ATGACTTTGGGATCCAGTCG	TGG AACCAGATGGGAAAGAG
**GS**	GACACTGAGCAGGGCTTTT	GGG CCTGGG ATACTTAAAGC
**LGP**	CCAGAGTGCTCTACCCCAAT	CCACAAAGTACTCCTGTTTCAGC
**GK**	TCCCTGTAAGGCACGAAGACAT	ATTGCCACCACATCCATCTCA
**CS**	TGCCCACACAAGCCATTTG	CTGACACGTCTTTGCCAACTT
**GLUT1**	CTTCTCTGTCGGCCTCTTTGT	ACAGCTCCAAGATGGTGACCT
**GLUT4**	TCGTCATTGGCATTCTGGTTG	AGCTCGTTCTACTAAGAGCAC
**ACO**	CAGCACTGGTCTCCGTCATG	CTCCGGACTACCATCCAAGATG
**CPT1a**	ACCCTGAGGCATCTATTGACAG	ATGACATACTCCCACAGATGGC
**MCAD**	GGC AAATGCCTGTGATTCTT	CCATTGCGATCTTGAAACCT
**SIRT1**	GACGCTGTGGCAGATTGTTA	GGAATCCCACAGGAGACAGA
**SIRT4**	CGCTGCTCAAGATCCCTAAG	GCGACACAGCTACTCCATCA
**SIRT6**	GGCTACGTGGATGAGGTGAT	GGCTCAGCCTTGAGTGCTAC

Figure S1. Effects of BDSW on the expression of sirtuin family genes in muscles (**A**) and livers (**B**) of mice fed the ND, HFD, and HFD with BDSW for 20 weeks. Each value represents the mean ± SEM (n = 8 per group). * $P < 0.05$, ** $P < 0.01$: Significant difference *vs.* HFD-fed group. ND, normal diet; HFD, high-fat diet.

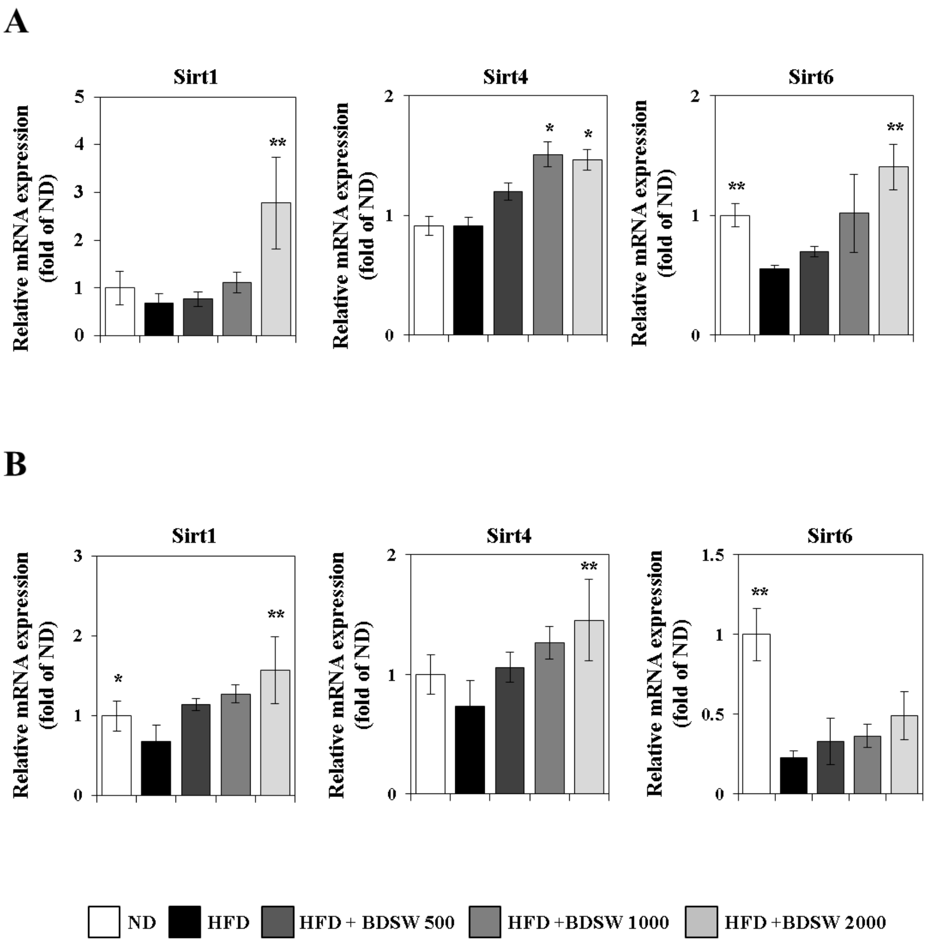

Supplement: Supplementary File 1 — Supplementary Information (PDF, 45 KB) [file marinedrugs-11-04193-s001.pdf]
